# Improving access to chimeric antigen receptor T-cells for refractory or relapsing diffuse large B cell lymphoma therapy in Asia

**DOI:** 10.1007/s12672-025-01860-5

**Published:** 2025-02-14

**Authors:** Ya Hwee Tan, Dok Hyun Yoon, Andrew J. Davies, Christian Buske, Yang Liang Boo, Nagavalli Somasundaram, Francesca Lim, Shin Yeu Ong, Anand Jeyasekharan, Koji Izutsu, Won Seog Kim, Jason Yongsheng Chan

**Affiliations:** 1 Division of Medical Oncology, National Cancer Centre Singapore, Singapore, Singapore; 2 Department of Oncology, Asan Medical Center, University of Ulsan College of Medicine, Seoul, South Korea; 3 School of Cancer Sciences, Faculty of Medicine, University of Southampton, Southampton, UK; 4 Institute of Experimental Cancer Research, University Hospital of Ulm, Ulm, Germany; 5 Department of Hematology, Hospital Sultanah Aminah, Johor Bahru, Malaysia; 6 Duke-NUS Medical School, National Cancer Centre Singapore, Singapore, Singapore; 7 Department of Haematology, Singapore General Hospital, Singapore, Singapore; 8 Department of Haematology-Oncology, National University Cancer Institute, Singapore, Singapore; 9 Cancer Science Institute of Singapore, National University of Singapore, Singapore, Singapore; 10 Department of Hematology, National Cancer Center Hospital, Tokyo, Japan; 11 Samsung Medical Center, Sungkyunkwan University School of Medicine, Seoul, South Korea

**Keywords:** CAR-T, Diffuse large B-cell lymphoma, Immunotherapy, Lymphoma, Novel therapy

## Abstract

Chimeric antigen receptor T-cell (CAR-T)-mediated therapies have shown promising clinical benefit in patients with refractory or relapsing (R/R) diffuse large B-cell lymphoma (DLBCL). However, CAR-T treatment presents challenges such as lack of drug accessibility, financial barriers, variable physician preference or experience, and risk assessment based on patient-specific characteristics. This article thus aims to provide an overview of the CAR-T landscape for R/R DLBCL in Asia, with a focus on identifying barriers to access, from the perspective of Asian and international lymphoma experts. Presently, existing clinical data indicate that CAR-T therapy is a potentially curative strategy for R/R DLBCL in addition to stem cell transplantation, provided the patient’s disease profile and treatment history have been thoroughly considered. However, longer-term follow-up data from large-scale studies are needed to confirm curative potential and define optimal sequencing of CAR-T in the context of novel emerging treatments, such as bi-specific antibodies, in the management of R/R DLBCL. Consequently, further research into CAR-T would benefit from collaboration between institutions. Furthermore, there is a wide disparity in CAR-T accessibility across regions due to complicated logistics and cost, which represent a significant barrier to patients in Asia. Hence, there is a need to increase representation and engagement across different stakeholders such as policymakers, payers, and the industry to arrive at a consensus on patient selection, establish clear guidelines, and develop strategies to lower CAR-T costs. Ultimately, data can support a multi-stakeholder approach when devising strategies to make CAR-T feasible and sustainable for patients.

## Introduction

Diffuse large B-cell lymphoma (DLBCL) is one of the most common subtypes of non-Hodgkin lymphoma (NHL) worldwide, with broad heterogeneity in disease biology and clinical outcomes [1–3]. Globally, NHL comprised 3.0% and 2.6% of malignancies in men and women, respectively [4]. Global incidence rates have also been observed to vary among different populations widely, including in Asia [4–6]. In 2020, NHL was two times higher in high or very high Human Development Index (HDI) countries (e.g., China, Japan, Singapore, and South Korea) compared with low or medium HDI countries [4]. Although global incidence of NHL has generally stabilised in recent years [4, 6, 7], some epidemiological studies have suggested that the number of new diagnoses is projected to increase by over 30 to 50% in high and very high HDI countries by 2040 [6, 7]. Furthermore, population-based studies and database reports in Asia suggest that the incidence of NHL has steadily and substantially increased over recent decades in countries including China, Japan, Singapore, and South Korea [8–11].

As approximately 30–40% of patients with DLBCL are refractory or relapsing (R/R) with first-line chemoimmunotherapy and two thirds will continue to relapse after second-line treatment (high-dose chemotherapy and autologous stem cell transplant [ASCT]) [1], there is a need for effective, safe, and accessible treatments for DLBCL beyond current frontline therapies. Chimeric antigen receptor T-cell (CAR-T)-mediated therapies are one such option, with trials showing promising clinical benefit and a positive impact on survival outcomes in DLBCL, with phase II/III trials reporting objective response rates (ORR) of 53–86% and progression-free survival (PFS) of 2.9–14.8 months [12–22]. However, patients and their clinicians often face challenges with using CAR-T in day-to-day practice despite its clinical benefits. Such barriers to treatment in a real-world setting include accessibility, complex or highly specific reimbursement policies, variable physician preference and experience, as well as patient-specific characteristics [3, 23].

To further understand the current landscape of R/R DLBCL management in Asia, a joint meeting comprising expert lymphoma experts was convened to discuss challenges to CAR-T use in R/R DLBCL, based on existing evidence and their clinical experience, as well as future strategies to optimise management and increase accessibility of CAR-T treatment for patients. In this paper, we review the current treatment landscape of CAR-T in R/R DLBCL and summarise the experts’ perspectives toward this topic.

## Methods

On December 1, 2023, a joint meeting took place at the National Cancer Centre Singapore, prior to the European Society for Medical Oncology Asia Congress 2023, involving major stakeholders in lymphoma care across Asia. A closed session was convened by an invited panel of lymphoma experts from Asia (Japan, Malaysia, Singapore, and South Korea) and internationally (Germany and the United Kingdom) to discuss on the current state of CAR-T therapy in R/R DLBCL, considering existing evidence and the panellists’ clinical experience, with a specific focus on identifying common barriers to access and discussing potential strategies to overcome them.

## Therapeutic landscape of approved treatments for R/R DLBCL

### United States (US) Food and Drug Administration (FDA) approved treatments

There has been an increase in the number of therapeutic agents available for R/R DLBCL in the past decade [1, 24]. The major clinical trials on therapeutic agents approved by the US FDA for R/R DLBCL are summarised in Table 1.

**Table 1:** Major clinical trials for R/R DLBCL

Drug class	Regimen (trial name); *reference*	Trial design, n^a^	Key inclusion criteria^a^	Median follow-up (months)^a^	Major efficacy endpoints^a^	US FDA approval^a^
ADC	Polatuzumab vedotin, bendamustine, rituximab; *Sehn *et al*. 2020, Sehn *et al*. 2022 *[26, 27]	Phase II, 106	≥ 1 line of therapy and transplant-ineligible	48.9	• ORR 41.5% (CR 38.7%) • Median OS 12.5 months • Median PFS 6.6 months	June 10, 2019
	Loncastuximab tesirine (LOTIS-2); *Caimi *et al*. 2021, Caimi *et al*. 2023 *[28, 29]	Phase II, 145	≥ 2 lines of therapy	7.8	• ORR 48.3% (CR 24.8%) • Median OS 9.5 months • Median PFS 4.9 months	April 23, 2021
Bispecific antibody	Epcoritamab (EPCORE NHL-1); *Hutchings *et al*. 2021, Thieblemont *et al*. 2023 *[33, 34]	Phase I/II, 157	≥ 2 lines of therapy	10.7	• ORR 63.1% (CR 38.9%) • Median OS not reached • Median PFS 4.4 months	May 19, 2023
	Glofitamab (NP30179); *Dickinson *et al*. 2022 *[32]	Phase I/II, 155	≥ 2 lines of therapy	12.6	• ORR 52% (CR 39%) • Median OS not reached • Median PFS 4.9 months	June 15, 2023
CAR-T	Axicabtagene ciloleucel (ZUMA-1); *Neelapu *et al*. 2017, Locke *et al*. 2019, Neelapu *et al*. 2023 *[16, 18, 19]	Phase II, 101	Refractory DLBCL, defined as progressive or stable disease as the best response to the most recent chemotherapy regimen or disease progression or relapse within 12 months after ASCT	63.1	• ORR 83% (CR 58%) • Median OS 25.8 months • Median PFS 5.9 months	October 18, 2017
	Tisagenlecleucel (JULIET); *Schuster *et al*. 2019, Schuster *et al*. 2021, Chong *et al*. 2021 *[14, 20, 21]	Phase II, 115	Ineligible for or had disease progression after ASCT	40.3	• ORR 53% (CR 39%) • Median OS 11.1 months • Median PFS 2.9 months	May 1, 2018
	Lisocabtagene maraleucel (TRANSCEND NHL 001); *Abramson *et al*. 2020, Abramson *et al*. 2024 *[12, 13]	Phase II, 270	≥ 2 lines of therapy	19.9	• ORR 73% (CR 53%) • Median OS 27.3 months • Median PFS 6.8 months	February 5, 2021
	Axicabtagene ciloleucel (ZUMA-7); *Locke *et al*. 2022, Westin *et al*. 2023 *[17, 22]	Phase III, 180	Refractory to or relapsed ≤ 12 months after first-line treatment	24.9^b^	• ORR 83% (CR 65%) • Median OS not reached^b^ • Median PFS 14.7 months^b^	April 1, 2022
	Lisocabtagene maraleucel (TRANSFORM); *Kamdar *et al*. 2022 *[15]	Phase III, 92	Refractory to or relapsed ≤ 12 months after first-line treatment	6.2	• ORR 86% (CR 66%) • Median OS not reached • Median PFS 14.8 months	June 24, 2022
Humanised Fc-modified cytolytic CD19 antibody	Tafasitamab, lenalidomide (L-MIND); *Salles *et al*. 2020; Duell *et al*. 2023 *[30, 31]	Phase II, 80	≥ 1 line of therapy and were transplant-ineligible	44.0	• ORR 57.5% (CR 41.3%) • Median OS 33.5 months • Median PFS 11.6 months	July 31, 2020
SINE	Selinexor (SADAL); *Kalakonda *et al*. 2020 *[35]	Phase II, 127	≥ 2 lines of therapy and progressed after or were transplant-ineligible	NR	• ORR 28% (CR 11.8%) • Median OS 9.1 months • Median PFS 3.5 months	June 22, 2020

^a^Data reported are from the latest trials for each drug

^b^Patients in an updated overall survival analysis at 5 years had a median follow-up of 47.2 months, with an estimated 4-year OS of 54.6% and estimated 4-year PFS of 41.8%[22]

*ADC* antibody–drug conjugate, *ASCT* autologous stem cell transplant, *CAR-T* chimeric antigen receptor T-cell, *CR* complete response, *DLBCL* diffuse large B cell lymphoma, *FDA* Food and Drug Administration, *NR* not reported, *ORR* objective response rate, *OS* overall survival, *PFS* progression-free survival, *R/R* refractory or relapsing, *SINE* selective inhibitors of nuclear export, *US* United States

#### CAR-T

To date, axicabtagene ciloleucel (axi-cel) [16–19, 22], lisocabtagene maraleucel (liso-cel) [12, 13, 15], and tisagenlecleucel (tisa-cel) [14, 20, 21] are the only three CAR-T drugs approved for use in adult R/R DLCBL patients who have received 2 or more prior lines of systemic therapy.

Recent data from a five-year follow-up of the phase II ZUMA-1 trial reported sustained overall and disease-specific survival with axi-cel, with an estimated five-year overall survival (OS) rate of 42.6% and disease-specific survival rate of 51.0%, supporting the curative potential of CAR-T therapy in R/R DLBCL [18]. Subsequently, axi-cel and liso-cel were both approved for patients with relapsed or refractory disease (within 1 year after first-line treatment) in the second-line setting [15, 22]. The phase III ZUMA-7 trial also demonstrated significantly longer overall and event-free survival of R/R DLBCL patients on axi-cel compared with standard care (chemoimmunotherapy, followed by high-dose chemotherapy with ASCT in patients with a response), with an estimated four-year OS of 54.6% versus 46.0%, respectively [17, 22]. TRANSFORM, a phase III trial in liso-cel, similarly reported improved median event-free survival in R/R DLBCL patients on liso-cel (10.1 months) compared with patients receiving standard care treatment (2.3 months) [15].

#### Other novel therapies

Other novel therapies used in R/R DLBCL patients include antibody–drug conjugates (ADC) such as polatuzumab vedotin, which can be used in combination with rituximab, cyclophosphamide, doxorubicin, and prednisone (pola-R-CHP) as first-line treatment [25]. Polatuzumab vedotin in combination with bendamustine and rituximab [26, 27] has also been investigated for patients ineligible for ASCT or CAR-T, alongside loncastuximab tesirine (ADC) [28, 29], and tafasitamab (humanised Fc-modified cytolytic CD19 antibody) in combination with lenalidomide [30, 31]. Bispecific antibodies are also an emerging novel strategy approved for patients with DLBCL, including glofitamab and epcoritamab [32–34]. Finally, selinexor, a first-in-class selective inhibitor of nuclear export (SINE), was first approved in R/R multiple myeloma and subsequently granted an indication extension to R/R DLBCL [35, 36].

### Current therapeutic landscape in Asia

Across Asia, anti-CD20 monoclonal antibody rituximab plus cyclophosphamide, doxorubicin, vincristine, prednisolone chemoimmunotherapy remains the current standard of care for most patients with DLBCL, consistent with international practice guidelines [1]. Alternative regimens such as pola-R-CHP and dose-adjusted EPOCH-R (etoposide, prednisone, vincristine, cyclophosphamide, doxorubicin, and rituximab) may also be considered for high-risk cases [37, 38]. Even in transplant-eligible patients with R/R disease relapsing within 12 months of first-line treatment, reinduction chemotherapy followed by high dose chemotherapy and then ASCT consolidation has been the preferred strategy, as CAR-T is presently neither reimbursed nor indicated.

Across Asia, disparities were noted in the availability of CAR-T for treatment of R/R DLBCL. In Japan, axi-cel, liso-cel, and tisa-cel are used in R/R DLBCL patients [39]. In Singapore, axi-cel and tisa-cel are currently indicated for R/R DLBCL [40, 41]; recent treatment advances in DLBCL and the practice landscape in Singapore were reviewed in Chan et al. 2023 [1]. In South Korea, prognosis of R/R DLBCL patients following frontline rituximab-based chemoimmunotherapy is dismal [42, 43]. Currently, R/R DLBCL patients in South Korea move to CAR-T after failing standard care [43]; tisa-cel is the only CAR-T currently available [44]. In contrast, South-East Asian countries such as Malaysia, Vietnam, and the Philippines were noted to have limited or no access to CAR-T. Patients in these countries can only access CAR-T through compassionate use programmes, highlighting an urgent need to improve patient access to novel therapeutic agents. In Asia, aside from CAR-T, other novel agents such as ADCs (except for polatuzumab vedotin), bispecific antibodies, and SINE are either not currently available or not reimbursed. For example, in Singapore, glofitamab and epcoritamab are available only on compassionate access and awaiting formal approval. The exact cost of these therapies however, is presently unclear. In South Korea, glofitamab and selinexor are approved, but not currently reimbursed.

### Expert consensus on the curative potential of CAR-T in the second or third-line setting for R/R DLBCL

Given the number of existing treatments and rapidly evolving therapeutic landscape of R/R DLBCL, it has become increasingly complex and challenging to decide on the optimal therapy after failing frontline treatment. Thus, a discussion about CAR-T as a curative treatment modality in the second or third-line setting for DLBCL is warranted.

#### Efficacy

CAR-T is currently a second-line option for R/R DLBCL (refractory/relapsed disease within 12 months of first-line chemoimmunotherapy), and has demonstrated efficacy in both second or third-line settings compared with non-CAR-T therapies [15, 17, 22]. A systematic literature review (SLR) and meta-analysis (n = 3 included studies) reported that CAR-T therapy at second-line showed superior outcomes compared with standard of care in R/R DLBCL patients [45]. Substantial survival benefit and durability of CAR-T response beyond the second-line setting compared with non-CAR-T salvage therapies have also been reported in comparisons of international clinical trials [46, 47]. A separate SLR and meta-analysis (n = 70 included studies) noted that R/R DLBCL patients treated with CAR-T demonstrated significantly better outcomes compared with patients treated with chemotherapy, but CAR-T did not outperform chemotherapy followed by ASCT in the second-line setting [48]. However, it was noted that the included studies on CAR-T (n = 5), all investigated efficacy beyond the second-line setting [12, 19, 20, 49, 50], and there have since been updated data supporting the efficacy of axi-cel and liso-cel in a second-line setting [15, 17, 22]. Crude grouping of treatment regimens in this SLR was noted as a limitation, due to heterogeneity of study characteristics, eligibility criteria, and treatment regimens of included studies [48].

Clinical trial data have also shown that clinical outcomes and risks of CAR-T toxicity may differ based on an individual patient’s characteristics or disease profile [23, 51]. Biomarkers, such as T-cell exhaustion, have been investigated in an effort to identify reliable predictors of CAR-T response [51]. For example, data suggest that T-cell exhaustion can limit CAR-T therapeutic efficacy, and strategies to circumvent this may help maintain efficacy of treatment [52]. Notably, markers of T-cell exhaustion such as expression of immune checkpoint molecules may be influenced by the patients’ disease phenotype [51], further highlighting the relevance of considering the individual clinical profile of patients.

Based on these international data, careful patient selection, with thorough consideration of the patient’s disease profile and treatment history, are key to optimising the long-term benefits of CAR-T in the second or third-line setting for R/R DLBCL. Proactive monitoring of biomarkers may also facilitate identification of patients who would have the highest chances of success on CAR-T, though further research is required into this area. Considering these factors, current data support CAR-T therapy as a potentially curative strategy for R/R DLBCL in addition to ASCT. However, longer-term follow-up data from large-scale studies and emergent data on bispecific antibodies are needed to define optimal sequencing of CAR-T in management of R/R DLBCL.

#### Safety

In our local practices, clinicians are increasingly proficient in identifying and managing adverse events associated with CAR-T toxicity, such as cytokine release syndrome (CRS) and immune effector cell-mediated neurotoxicity syndrome (ICANS). Consequently, safety concerns are generally not a significant barrier to CAR-T use. However, careful patient selection remains important in managing the risk of toxicity, as significant challenges in managing severe cases of toxicity, such as profound CRS, capillary leak syndrome, and/or multiorgan failure, can still arise. Concerns about longer-term adverse events such as cytopenia, B-cell aplasia with attendant hypogammaglobulinemia, significant infection risks, and possibility of secondary malignancies should also be considered. Given the role of multidisciplinary care in disease management, staff at reinfusion centres also require careful training in the recognition and management of these CAR-T associated adverse events.

#### Risk stratification of patients

Factors reported to influence durability of remission after CAR-T in R/R B-cell lymphoma patients include depth of response, malignancy type, tumour burden and location, receipt of previous lymphodepleting chemotherapy, and peak CAR-T cell levels [53]. Patients with a high disease burden (e.g., rapidly progressive disease), active infections, low platelet count, or high serum lactate dehydrogenase levels were also noted to be high-risk features for severe adverse events and increased morbidity and mortality [54]. This is in alignment with patient/disease characteristics observed in local practice to be associated with poor CAR-T response and outcomes in practice, including high disease burden (e.g., high tumour volume), aggressive disease, history of being unresponsive to other treatments (e.g., chemotherapy or gene therapy), or symptomatic patients. Conversely, patients receiving CAR-T while in disease remission typically benefit in the long term.

CD5 expression is a potential independent poor prognostic factor of DLBCL in Asia. There is evidence of a higher prevalence of CD5-positive DLBCL patients in Japan versus Western countries [55]. Studies have also shown that CD5-positive patients with DLBCL in Asia have inferior survival tendency [56], or poor survival outcomes following front-line chemotherapy [57, 58]. However, the prognostic value of CD5 may only apply to patients with unique clinicopathologic features [59], and its exact mechanism remains uncertain. Further research on the clinical outcomes of CAR-T in patients with varying CD5 expression can aid in developing CD5-optimised CAR-T for DLBCL.

Consequently, it is vital to optimise patient selection for CAR-T to maximise curative potential of treatment, as well as avoid or minimise short-term and long-term toxicities.

#### Bispecific antibodies as an alternative to CAR-T in the third-line setting for R/R DLBCL

There are several advantages to bispecific antibodies, including the ability to deliver these treatments outside academic centres, bridging to CAR-T, and having a shorter turnaround time compared with CAR-T [44, 60]. A recent meta-analysis showed that CAR-T had superior efficacy to bispecific antibodies as a third- or later-line DLBCL treatment, with higher complete response and progression-free survival rates; however, CAR-T exhibited a greater incidence of grade ≥ 3 adverse events [61]. More data on long-term safety and their associated cost implications are therefore required. Furthermore, a lack of long-term data for bispecific antibodies was cited as a key limitation to their use. Current data do not strongly suggest that bispecific antibodies are curative in a third-line setting; for example, phase II trial data on glofitamab in patients with R/R DLBCL demonstrated that 37% of treated patients maintained progression-free survival at 12 months [32], but there are presently no data with longer follow-up available for glofitamab.

Current literature may therefore imply that bispecific antibodies provide disease control rather than being curative. Thus, there is a need for longer-term follow-up data to adequately assess their curative potential in R/R DLBCL patients receiving bispecific antibodies, alongside risks of adverse events and long-term complications such as infection.

## Accessibility barriers to CAR-T in R/R DLBCL

In addition to efficacy and safety concerns, the financial burden that new treatment modalities place on patients and the healthcare system is an important consideration in devising strategies aiming to increase CAR-T accessibility to patients. A cost-effectiveness analysis of axi-cel and liso-cel as second-line therapy in DLBCL acknowledged that CAR-T improved clinical outcomes incrementally, but costs need to be substantially lowered for treatment to be cost-effective at a willingness-to-pay threshold of USD$200,000 per quality-adjusted life-year [62]. Findings from modelling studies in Singapore have been mixed in demonstrating CAR-T as a cost-effective option versus salvage chemotherapy in patients with R/R DLBCL who have failed ≥ 2 lines of systematic therapies [63, 64]. A recent systematic literature review extensively summarised other economic evaluations of CAR-T, revealing that while CAR-T is more costly than its comparators, the cost-effectiveness estimates remain uncertain [65]. Some countries have implemented financing schemes to alleviate cancer treatment costs and ensure cancer treatment costs and insurance premiums continue to remain affordable over time. For example, in Singapore, the Ministry of Health introduced a Cancer Drug List (CDL) that incorporates limited co-payment together with broad accessibility to approved cancer therapies. While this includes most of the standard approved therapies for lymphoma, treatments that are not listed on the CDL are otherwise not readily eligible for reimbursement [66].

### Expert consensus on barriers to CAR-T access

#### Affordability

Globally and in Asia, cost of CAR-T is a significant barrier for patient access [1, 3, 23, 44]. Across Asia, CAR-T also presents significant demand and unique challenges on the healthcare infrastructure. For example, unlike autografts which can be planned, patients requiring CAR-T may need immediate treatment representing a challenge to anticipate and allocate resource to such cases. Management of complications such as cytopenia, hypogammaglobinaemia, and infections in an outpatient setting post-discharge can also be logistically complex. In Singapore, for example, CAR-T is not reimbursed and insurance authorisation is a time-consuming process. Consequently, privately insured patients may also experience challenges in accessing CAR-T. Specifically, local insurance companies were noted to be less engaged than industry representatives in addressing financial barriers to CAR-T; despite industry assistance with funding, the cost of CAR-T remains unfeasible for some patients. Consequently, clinicians can also face difficulties when conducting financial counselling with patients on costs associated with CAR-T. Locally, improvements to the infrastructure are required to address these issues. In South Korea, reimbursement for each case is established by the Health Insurance Review and Assessment Service (HIRA), the national reimbursement agency, solely following CAR T-cell infusion. Challenges ensue when HIRA contests physicians regarding the appropriateness of the indication, potentially resulting in HIRA rejecting reimbursement and thereby imposing a notable financial burden on hospitals or physicians. These reimbursement hurdles, compounded by the inherent expenses linked with CAR-T therapy, present supplementary barriers to its widespread adoption in South Korea.

Compassionate use programmes and academic CAR-T therapies can facilitate patient access to CAR-T. However, each comes with their own set of challenges. Ensuring sustainability of accessing CAR-T through compassionate use programmes is a foreseeable challenge if CAR-T is elevated to a standard of care treatment. Academic CAR-T therapies can face complex issues, such as patency rights and product registration for use worldwide, which have implications on the accessibility and cost of these therapies. In Europe, the Hospital Exemption Clause enables manufacturing of advanced therapy medicinal products (including CAR-T) through academic platforms [67]. This legislation has facilitated access to innovative therapies in Europe [67], thereby highlighting the opportunities made possible by partnership between academia and the industry, which Asia could seek to adapt.

#### Geographic disparities

The number of CAR-T centres in a given country could be a barrier in delivering CAR-T to patients. For example, there are > 40 sites in Japan (of which 8 provide axi-cel, the remaining provide tisa-cel) and 11 sites in the United Kingdom that provide CAR-T for patients across the country. However, there is a geographic disparity in where these centres are situated. Patients may also be unwilling or unable to travel long distances to receive treatment, thereby contributing to inequities and geographic health disparities in receiving CAR-T.

## Expert consensus on strategies and solutions to challenges in delivering CAR-T

### Developing guidelines for patient selection

Consensus on patient selection guidelines would support clinicians in identifying patients who will derive the best medical benefit from CAR-T in the long-term, thereby guiding risk management (maximising clinical benefit while minimising risk of toxicity). Guidelines would inform standard operating procedures, accessibility protocols, and support the multidisciplinary team (MDT) in navigating complex cases to holistically weigh the benefits of CAR-T against treatment risks and resource required (e.g., cost and manpower). For example, patients who are young are often willing to risk the adverse effects of treatment rather than accepting palliative care. Such patients may benefit from thorough counselling on the risks of CAR-T to set expectations on treatment. Similarly, it may be important for clinicians to consider the remaining benefit of treatment against resource required in managing patients who are approaching the end of their disease-free survival period, and counsel such patients accordingly. The intensive care unit MDT would also benefit from streamlined guidance to efficiently manage severe, complex cases with complications, given the team can expend significant resources supporting such patients.

Other considerations in a patient selection guideline could include suitability of other novel therapies (e.g., bispecific antibodies or ADCs), as these drugs may fulfil unique roles in the treatment pathway for R/R DLBCL patients where CAR-T may not be appropriate. Research into biomarkers of CAR-T response could also complement guidelines for patient selection. In the absence of establishing such biomarkers, proactively monitoring patients on CAR-T could help identify those with ‘higher risk’ disease characteristics (e.g., symptomatic, high tumour volume). Such patients can then be managed and closely monitored based on their individual disease profile. Notably, long-term data on CAR-T safety outcomes would also inform the development of guidelines that are tailored to the patient demographic in Asia. Given the complexities of long-term follow-up in patients, local centres may consider real-world studies including analysing data from registries.

Beyond an in-patient setting, guidelines would also support continuity of patient care in outpatient settings (e.g., post-discharge care and management of complications). Guidance on risk assessment should also be underpinned by a high-quality management system that recognises challenges in patient coordination across different settings throughout their treatment journey. Importantly, these guidelines should be tailored to fit local clinical needs and management practices, and an example is Singapore’s technology guidance for tisa-cel [68, 69]. This can be achieved by forming local multidisciplinary teams that not only shape and advocate for the guidelines but also educate those who apply it in practice. Overall, funding will be a key requirement in developing guidelines for patient selection and risk management in Asia.

### Lowering financial barriers to CAR-T

CAR-T could present a financial challenge for patients due to its expensive manufacturing process and its accumulative financial burden throughout treatment and subsequent outpatient care [69, 70]. Innovative manufacturing approaches can therefore help to lower the cost of CAR-T and facilitate its integration into standard clinical management [69]. Patients should also have ongoing support and resources to manage the financial strain of completing their CAR-T regimen [70].

In Asia, there is a need to increase representation and engagement across different stakeholders (e.g., industry, clinicians, policymakers) to arrive at a consensus on patient selection and develop strategies to lower financial barriers to CAR-T for patients. In devising these strategies, it will be important to consider that definitions of affordability can also differ between the healthcare systems of different countries across Asia. Emphasis should also be placed on generating data, which would contribute towards cost-effectiveness assessments. Moreover, given the uncertainty in current CAR-T cost-effectiveness estimates, which are influenced by factors such as patient population and model assumptions [65], it is essential to conduct nuanced economic evaluations to assess its value across diverse patient groups. In line with results of such assessments, policymakers should be encouraged to consider the value of prioritising budget for CAR-T compared with other therapies that may not necessarily offer better efficacy outcomes than CAR-T. Appealing to policymakers to approve CAR-T as standard therapy may motivate insurance companies and the industry to bridge the financial gap for patients. Additionally, insurance policies should also recognise the true cost of the full CAR-T patient experience, including outpatient costs in the long-term [70].

Countries in Asia can also look to adapt the funding mechanisms that have been successful elsewhere. For example, adapting the ‘full/primary public pay’ models used in Australia [71, 72], Japan and Korea [73], or risk-sharing model (between manufacturers and public payers) in China [73]. Countries may also gain insights from observing Singapore’s ongoing efforts to establish a sustainable financing framework using national funding to improve patient access to CAR-T [74], and the unique hub-and-spoke model of optimising patient management in Southeast and South Asia [75].

### Collaboration and shared learning between institutions

It is important for clinicians to discuss and share learnings on CAR-T. As not all countries in Asia have access to CAR-T, mutual support and exchange of information is crucial in facilitating delivery of CAR-T to patients. Specifically, centralised selection processes can be valuable platforms to exchange information and learnings, which can include perspectives from multiple stakeholders. Internationally and in Asia, some countries have a centralised selection process for receipt of CAR-T, though the exact size and scope of this initiative varies. In the United Kingdom, patients are approved to receive CAR-T via a national CAR-T panel under the National Health Service, comprising patient representatives and representatives from CAR-T and non-CAR-T centres. In Singapore, combined lymphoma meetings are held by certain clinics to identify suitable patients. Institutions in Asia can also benefit from adapting models for training and patient selection from other countries (e.g., Australia and the United Kingdom).

## Conclusion

**Fig. 1:**
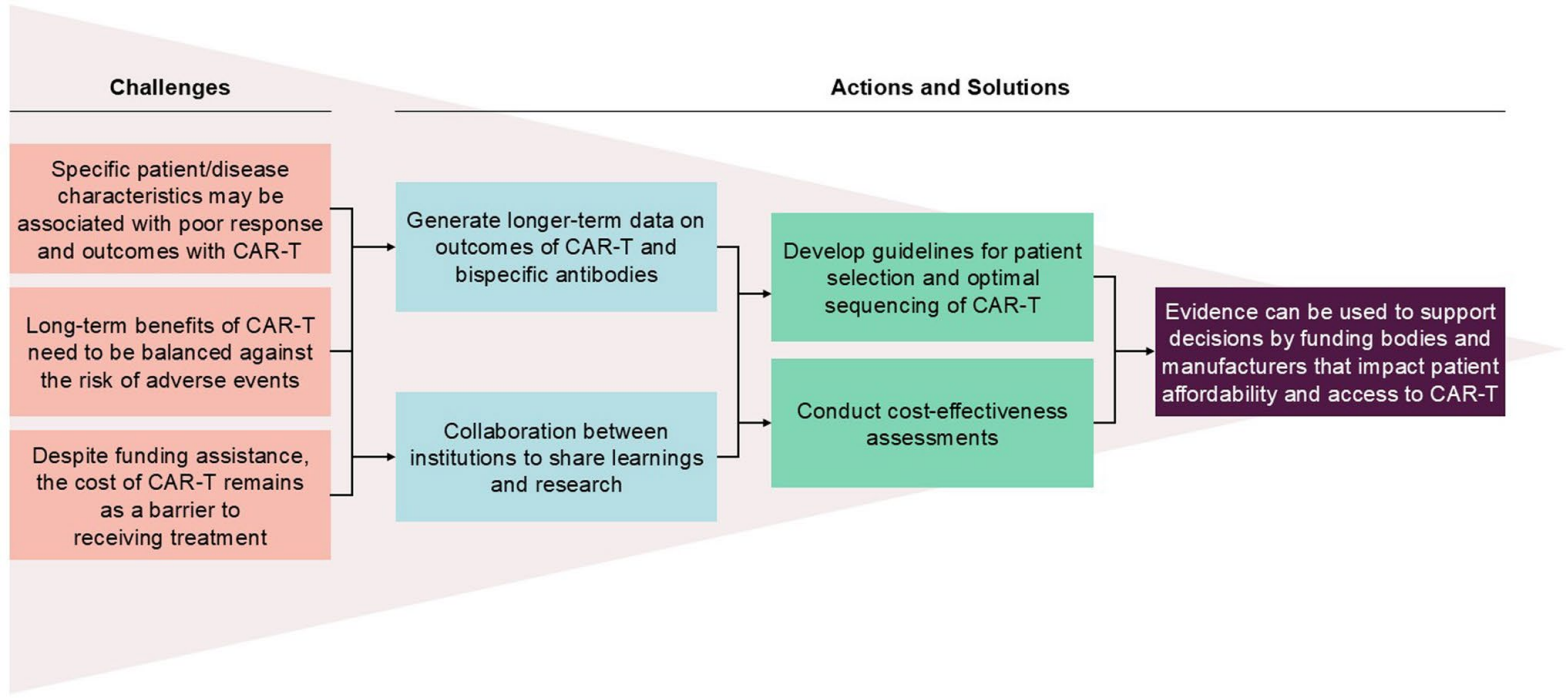
Summary of barriers to CAR-T treatment in Asia and potential solutions. The figure presents a visual summary of barriers to CAR-T treatment in Asia and potential solutions. Moving from the left to the right of the figure, each point is presented in a rectangle box to show how challenges map on to action points for evidence-generating activities, and how these data can be used to encourage policymakers and payers to bridge the financial gap between patients and CAR-T. On the far left-hand side, there are three boxes that summarise the challenges of CAR-T treatment in Asia, such as how: 1. specific patient/disease characteristics may be associated with poor response and outcomes with CAR-T, 2. long-term benefits of CAR-T need to be balanced against the risk of adverse events, and 3. despite funding assistance, the cost of CAR-T remains as a barrier to receiving treatment. These challenges link to two boxes on the right, which show the ways that these challenges can be collectively addressed, specifically by: 1. generating longer-term data on outcomes of CAR-T and bispecific antibodies, and 2. collaboration between institutions to share learnings and research. This continues on to two boxes on the further right, which show the collective practical applications and outputs of these learnings and data, specifically to: 1. develop guidelines for patient selection and optimal sequencing of CAR-T, and 2. conduct cost-effectiveness assessments. Finally, the box on the extreme right concludes that: evidence can be used to encourage policymakers and payers to bridge the financial gap between patients and CAR-T. *CAR-T* chimeric antigen receptor T-cell

The R/R DLCBL therapeutic landscape is rapidly evolving and emerging novel strategies continually add to the complexity of managing these patients. Advancements such as CAR-T offer patients more options than the previous decade, but can come with notable clinical and logistical challenges. The challenges associated with CAR-T in Asia and potential solutions are summarised in Fig. 1. In particular, the logistical challenges and high manufacturing cost of CAR-T remains one of the greatest challenges to expanding patient access.

Therefore, a multi-pronged approach to improving CAR-T is required to uplift the standard of care among R/R DLBCL patients in Asia. Firstly, data generating activities to establish longer-term outcomes of CAR-T would benefit from collaboration between institutions in Asia and internationally, as further research and consensus is required to establish clear guidelines for patient selection. These data can then support stakeholders, such as policymakers, payers, and the industry, in devising strategies to increase financial accessibility and sustainability of CAR-T for patients with R/R DLBCL in Asia.

## Data Availability

No datasets were generated or analysed during the current study.
